# Association between life’s essential 8 and testosterone deficiency in US men: findings from national health and nutrition examination survey (NHANES) 2011–2016

**DOI:** 10.3389/fendo.2024.1395576

**Published:** 2024-06-24

**Authors:** Yangyang Mei, Nuo Ji, Bo Zhang, Wei Xia, Xingliang Feng, Renfang Xu, Dong Xue

**Affiliations:** ^1^ Department of Urology, Jiangyin People’s Hospital of Jiangsu Province, Jiangyin, Jiangsu, China; ^2^ Department of Urology, The Third Affiliated Hospital of Soochow University, The First People’s Hospital of Changzhou, Changzhou, Jiangsu, China

**Keywords:** life’s essential 8, testosterone deficiency, NHANES, cardiovascular health, cross-sectional study

## Abstract

**Background:**

Testosterone deficiency (TD) is closely associated with cardiovascular diseases (CVD). We intended to explore the association of Life’s Essential 8 (LE8), the recently updated measurement of cardiovascular health, with the prevalence of TD among US male adults.

**Methods:**

The population-based cross-sectional study selected male adults aged 20 years or older from the National Health and Nutrition Examination Survey (NHANES) from 2011 to 2016. According to the American Heart Association definitions, the LE8 score was measured on a scale of 0–100, and divided into health behavior and health factor scores, simultaneously. Furthermore, these scores were categorized into low (0–49), moderate (50–79), and high (80–100) classifications. TD is defined as a total testosterone level below 300ng/dL. Correlations were investigated by weighted multivariable logistic regression, and the robustness of the results were verified by subgroup analysis.

**Results:**

A total of 4971 male adults with an average age of 47.46 ± 0.41 years were eligible for the final analyses, of whom 1372 were determined to have TD. The weighted mean LE8 score of the study population was 68.11 ± 0.41. After fully adjusting potential confounders, higher LE8 scores were significantly associated with low risk of TD (odd ratio [OR] for each 10-point increase, 0.79; 95% CI, 0.71–0.88) in a linear dose-response relationship. Similar patterns were also identified in the association of health factor scores with TD (OR for each 10-point increase, 0.74; 95% CI, 0.66–0.83). These results persisted when LE8 and health factor scores was categorized into low, moderate, and high groups. The inversed association of LE8 classifications and TD remained statistically significant among older, obese, and men without CVD.

**Conclusions:**

LE8 and its health factor subscales scores were negatively associated with the presence of TD in linear fashions. Promoting adherence to optimal cardiovascular health levels may be advantageous to alleviate the burden of TD.

## 1 Introduction

Testosterone is an indispensable male sex hormone primarily secreted by the Leydig cells in the testis and is mainly regulated by negative feedback of the hypothalamic-pituitary-gonadal axis (HPGA) (1). In addition to essential roles in male reproduction and sexual function (2), normal testosterone level also plays multiple necessary regulatory roles in cardiovascular function and recognitive function (3–5). Normal testosterone levels in men is defined between 300 and 1000 ng/dL, whereas testosterone deficiency (TD) is defined below 300 ng/dL accompanied with specific symptoms (6). Notably, TD afflicted 20% to 50% of men in the United States (US), while approximately 500000 individuals are newly diagnosed with TD annually (7, 8). In addition to the most common sexual symptoms like libido decline or erectile dysfunction, TD may lead to various nonsexual symptoms such as reduced energy, impaired concentration, and depressed mood (9, 10). It is worth noting that accumulated evidence has indicated tight association between TD and cardiovascular diseases (CVD) (11, 12). However, little research has been conducted on whether maintaining cardiovascular health (CVH) can elevate testosterone levels and reduce the incidence of TD.

To promote CVH of general population, the American Heart Association (AHA) initially introduced Life’s Simple 7 (LS7) as a metric to evaluate CVH, incorporating 3 health behaviors (diet, physical activity, and exposure to cigarette smoking) and 4 health factors (body mass index, fasting blood glucose, total cholesterol, and blood pressure), each metric classified as poor, intermediate, or ideal based in generally accepted clinical thresholds (13). However, the limitations of LS7 score were acknowledged during its usage. The LS7 does not account for the underlying social determinants of health and the context of mental health, both of which are considered crucial factors in enhancing and maintaining CVH (13). Additionally, the original definition of each LS7 component may not adequately capture the interindividual variation and intraindividual variability (14). Hence, the Life’s Essential 8 (LE8) was introduced to overcome the acknowledged limitations of LS7. The LE8 integrates sleep as an eighth metric, emphasizing mental health to enhance CVH, and updates the scoring of CVH metrics to increase sensitivity to interindividual differences and intraindividual variation (15, 16). Given the close association between TD and CVD, promoting LE8 can be an anticipatory and strategic measure to elevate testosterone level and alleviate the burden of TD. Nevertheless, no study has investigated the association between the LE8 score and TD to date.

Therefore, to fill this gap and add to the evidence on the relationship between LE8 and TD, we leverage nationally representative data from the 2011–2016 National Health and Nutrition Examination Survey (NHANES) to explore the association between the LE8 score and TD among US male adults. We hypothesize that a higher LE8 score will be significantly associated with a lower incidence of TD.

## 2 Materials and methods

### 2.1 Data source and study population

NHANES, a national and ongoing series of cross-sectional surveys, is overseen by the National Center for Health Statistics (NCHS) at the US Centers for Disease Control and Prevention (CDC). The NHANES, conducted in a 2-year cycle, is intended to evaluate the health and nutritional status of the U.S. population by combining personal interviews with physical examinations and laboratory tests. NHANES employed a sophisticated multiperiod probability-based sampling methods to obtain nationally representative sample for the civilian noninstitutionalized U.S. population. All study protocols were reviewed and approved by the research ethics review broad of NCHS, and each participant provided written informed consent.

**Figure 1:**
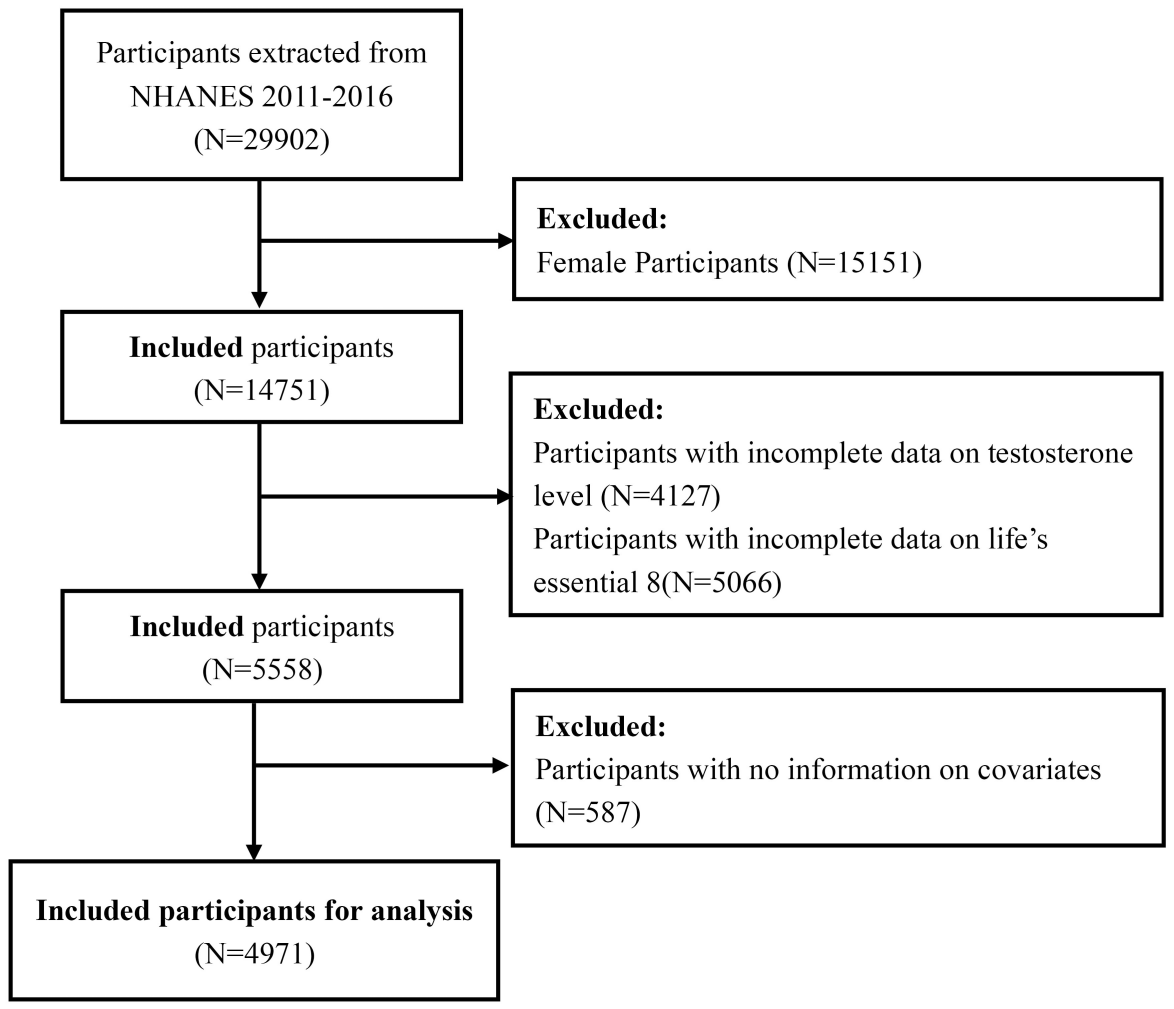
Flow chart of the selection process for study population.

Our study population was limited to three data cycles from 2011 to 2016 since only they measured testosterone levels and evaluated LE8 simultaneously. After excluding female participants, male participants aged <20 years, and those with missing data on testosterone level, LE8 metrics, and potential covariates from a total of 29902 participants in the selected NHANES cycles, the cohort finally involved 4971 adult male participants. The detailed sample selection process is displayed in **Figure 1**.

### 2.2 Exposure and outcome definitions

The LE8 CVH score is calculated based on 8 components: diet quality, physical activity duration, smoking status, sleep duration, body mass index, blood lipids, blood glucose, and blood pressure (14). The Healthy Eating Index (HEI) 2015, measured through subjects’ 24-hour dietary recall, was utilized to assess the diet metric (17). Information on physical activity (minutes of moderate or intense weekly exercise), tobacco/nicotine exposure (active tobacco consumption and exposure to secondhand smoke), sleep health (total sleep hours), diabetes, and medication history were obtained by self-reported questionnaires. Height, weight, and blood pressure were assessed during physical examinations, with BMI calculated as weight (in kilograms) divided by the square of height (in meters). Blood samples were collected and sent to central laboratories for analyzing blood lipids, plasma glucose, and hemoglobin A1c. Each of these components is scored on a scale from 0 to 100 based on its own set of scoring standards (14). The detailed algorithms for calculating LE8 scores for each metric in NHANES data could be found in **Supplementary Table S1**. The total LE8 score was calculated as an unweighted average of the 8 indicators (18). Meanwhile, participants were categorized as having high CVH with LE8 scores of 80–100, moderate CVH with scores of 50–79, and low CVH with scores of 0–49 (18). To further investigate the association between LE8 subscales and TD, this study employed equal definitions and cut-off points to categorize LE8 subscale scores as health behavior and health factor scores.

To minimize biological variability, morning serum specimens were collected following overnight fasting. After that, serum, separated from red cells within 6 hours of collection, underwent processing and storage before being shipped to the Division of Environmental Health Laboratory Sciences at the National Center for Environmental Health, CDC, for analysis. Specimen collection and processing adhered to the guidelines provided in the NHANES Laboratory Procedures Manual (LPM). Serum testosterone was measured using the isotope dilution liquid chromatography tandem mass spectrometry (ID-LC-MS/MS), following the National Institute for Standards and Technology’s (NIST) reference method. The assay had a detection limit of 0.75 ng/dL and a reportable range of 2.5–1000 ng/dL. TD, the outcome of interest in our study, was defined as a serum testosterone level <300 ng/dL according to American Urological Association guidelines (6).

### 2.3 Study covariates

In this study, demographic covariates included age, BMI, race/ethnicity, educational levels, marital status, and poverty to income ratio. Health habits and conditions covariates included smoking status, alcohol intake, hypertension, diabetes mellitus, CVD, and hyperlipidemia. Furthermore, some biochemical indicators that may potentially influence testosterone levels were also considered as potential covariates, including uric acid (UA), total cholesterol (TC), and high-density lipoprotein (HDL). Age was categorized into three groups: 20 to 40 years, 40 to 60 years, and ≥60 years. Meanwhile, the BMI was also separated into 3 categories: <25 kg/m^2^, 25–30 kg/m^2^, and ≥30 kg/m^2^. Race and ethnicity were categorized as Mexican American, Non-Hispanic White, Non-Hispanic Black, Other Hispanic, and Other Race. Education levels were divided into three groups: below high school, high school, and above high school. Living alone or living with partner were the 2 marital status categories. The PIR was classified into 3 groups: <1.3, 1.3–3.5, and ≥3.5.

Smoking status was recognized based on participants’ responses to questions regarding whether they had smoked at least 100 cigarettes during their lifetime and if they were currently smoking. Participants responding “Yes” to both questions were considered “Current smokers”, while those responding “No” to both questions were considered “Never smokers”. The remaining participants were categorized as “Former smokers”. Alcohol intake was classified into five categories: no alcohol consumption (<12 drinks/lifetime), former alcohol consumption (≥12 drinks/lifetime and no drink now), mild alcohol consumption (2 drinks per day), moderate alcohol consumption (3 drinks per day), and heavy alcohol consumption (>3 drinks per day) (19). Hypertension was defined as a mean of multiple blood pressure measurements ≥140/90 mmHg, a prior diagnosis of hypertension, or the prescription of hypertension-lowering medication (20). Diabetes was defined as a fasting serum glucose greater than 126 mg/dL, HbA1c ≥6.5%, plasma glucose level>200 mg/dL at 2 hours after an oral glucose tolerance test (OGTT), taking diabetic medications, or being diagnosed by a physician with diabetes (21). CVD was defined as a prior diagnosis of congestive heart failure, coronary heart disease, angina, or heart attack. Hyperlipidemia was defined as a total cholesterol > 240 mg/dL, taking hypercholesterolemia medications, or being diagnosed with high cholesterol by a physician (22).

### 2.4 Statistical analyses

All statistical analyses strictly followed the NHANES analytic reporting guidelines, which account for the intricate survey design by assigning weights to participants to correct for oversampling of specific subgroups (23). Therefore, the sample weights from three continuous cycles were appropriately combined using the method recommended by CDC, and the combined sample weights were taken into account when making comparisons and building regression models. The study population were divided into two groups based on the presence of TD. The continuous variables were presented as weighted mean and standard error (SE), while the categorical variables were presented as weighted percentage. The difference in continuous variables between groups were compared using a linear regression model, while the difference in categorical variables were compared using a weighted chi-square test. Survey-weighted univariable and multivariable logistic regressions were conducted to investigate the independent association between LE8 score and its subscales with TD. The results were presented as odds ratios (OR) with corresponding 95% confidence intervals (CI). No potential covariates were adjusted in Model 1. In Model 2, age, race, marital status, educational level, and PIR were adjusted. In Model 3, the remaining potential variables, including BMI, smoking status, alcohol intake, hypertension, diabetes mellitus, CVD, hyperlipidemia, TC, HDL, and UA were all fully adjusted based on Model 2. In all regression models, the exposure variables (LE8 score, health behavior score, and health factor score) were considered as both continuous and categorical variables (high classification, moderate classification, and low classification). Additionally, the smooth curve fitting and generalized additive model based on Model 3, were employed to explore any non-linear relationship between significant exposure variables with TD.

To further explore the effect of confounders on the associations between LE8 and TD, subgroup analysis, based on Model 3 were performed considering age, BMI, marital status, smoking status, and history of hypertension, diabetes mellitus, CVD, and hyperlipidemia. P-values for interactions were determined using the log-likelihood ratio test. Likewise, the LE8 score was considered as both continuous and categorical variables. All statistical analyses were performed on R 4.0.2 with R packages, and EmpowerStats (http://www.empowerstats.com, X&Y Solutions, Inc.). Differences were considered statistically significant when the two-sided p-value was <0.05.

## 3 Results

### 3.1 Baseline characteristics of study population

Ultimately, a total of 4971 male participants aged 20 years or older were included. Baseline characteristics of the study population were summarized by the category of TD status in **Table 1**. The weighted mean age of the study population was 47.46 ± 0.41 years, and 26.53% of participants aged older than 60 years old. The mean LE8 score was 68.11 ± 0.41 and the weighted percentages of low, moderate, and high CVH were 9.21%, 68.20%, and 22.59%, separately. Among the study population, 1372 participants were diagnosed with TD. Populations with TD were older (50.37 ± 0.62 years vs. 46.39 ± 0.50 years), more likely to be obese (31.99 ± 0.28 kg/m^2^ vs. 27.75 ± 0.13 kg/m^2^), living with partner, and had higher prevalence rate of hypertension, diabetes mellitus, CVD, and hyperlipidemia versus those with non-TD. Compared with individuals with TD, participants without TD had higher scores in LE8, health behaviors, and health factors. However, the contrast in health factors between the two groups (72.33 ± 0.51 vs. 60.41 ± 0.56) is more pronounced than that observed in health behaviors (67.57 ± 0.50 vs. 65.80 ± 0.80).

**Table 1:** Baseline characteristics of study population from 2011–2016 National Health and Nutrition Examination Survey (NHANES), divided by presence of testosterone deficiency, weighted.

Characteristics	Total	Testosterone deficiency		P value
		No (N=3599)	Yes (N=1372)	
Age, years	47.46 ± 0.41	46.39 ± 0.50	50.37 ± 0.62	< 0.0001
20–40y, %	35.70	38.65	27.67	
40–60y, %	37.77	36.35	41.62	
≥60y, %	26.53	24.99	30.71	
BMI, kg/m^2^	28.89 ± 0.13	27.75 ± 0.13	31.99 ± 0.28	< 0.0001
<25kg/m^2^, %	25.62	30.83	11.43	
25–30kg/m^2^, %	38.48	40.45	33.09	
≥30kg/m^2^, %	35.91	28.72	55.48	
PIR	3.13 ± 0.07	3.11 ± 0.07	3.20 ± 0.08	0.19
<1.3, %	19.40	19.72	18.53	
1.3–3.5, %	34.64	35.09	33.41	
≥3.5, %	45.96	45.19	48.06	
Race, %				0.13
NH-black	8.93	9.45	7.51	
NH-white	70.61	69.95	72.38	
Mexican American	8.06	8.04	8.10	
Other Hispanics	5.22	5.36	4.86	
Other races	7.18	7.20	7.15	
Level of education, %				0.90
Below high school	13.40	13.34	13.57	
High school	21.97	22.14	21.50	
Above high school	64.64	64.53	64.94	
Marital status, %				< 0.0001
Living alone	31.63	34.26	24.49	
Living with partner	68.37	65.74	75.51	
Smoking, %				< 0.001
Never	50.28	50.98	48.39	
Former	29.63	27.40	35.72	
Current	20.09	21.63	15.89	
Alcohol status, %				0.002
Never	6.87	6.56	7.73	
Former	14.66	13.12	18.86	
Mild	42.06	42.26	41.49	
Moderate	12.50	13.01	11.12	
Heavy	23.91	25.05	20.80	
Hypertension, %				< 0.0001
No	60.79	64.14	51.68	
Yes	39.21	35.86	48.32	
Diabetes mellitus, %				< 0.0001
No	86.08	88.79	78.70	
Yes	13.92	11.21	21.30	
Cardiovascular disease, %				< 0.0001
No	90.39	91.43	87.55	
Yes	9.61	8.57	12.45	
Hyperlipidemia, %				< 0.0001
No	31.01	35.25	19.49	
Yes	68.99	64.75	80.51	
UA, mg/dL	6.04 ± 0.03	5.94 ± 0.03	6.32 ± 0.05	< 0.0001
TC, mg/dL	188.83 ± 1.00	188.55 ± 1.06	189.59 ± 1.63	0.53
HDL, mg/dL	48.26 ± 0.33	50.00 ± 0.44	43.53 ± 0.45	< 0.0001
Total testosterone, ng/dl	414.91 ± 3.51	484.46 ± 3.62	225.53 ± 1.71	< 0.0001
LE8 score	68.11 ± 0.41	69.95 ± 0.43	63.11 ± 0.58	< 0.0001
LE8 (CVH), %				< 0.0001
Low (0–49)	9.21	7.13	14.86	
Moderate (50–79)	68.20	66.22	73.58	
High (80–100)	22.59	26.65	11.56	
Health behaviors score	67.09 ± 0.49	67.57 ± 0.50	65.80 ± 0.80	0.03
Health behaviors score, %				0.01
Low (0–49)	17.65	17.48	18.12	
Moderate (50–79)	51.84	50.33	55.93	
High (80–100)	30.51	32.19	25.95	
Health factors score	69.12 ± 0.44	72.33 ± 0.51	60.41 ± 0.56	< 0.0001
Health factors score, %				< 0.0001
Low (0–49)	14.44	10.42	25.37	
Moderate (50–79)	53.75	51.15	60.84	
High (80–100)	31.81	38.43	13.78	

BMI, body mass index; PIR, poverty income ratio; UA, uric acid; TC, total cholesterol; HDL, high-density lipoprotein; LE8, life’s essential 8; CVH, cardiovascular health.

Data are presented as weighted mean (SE) for continuous variables and unweighted frequencies (weighted percentages) for categorical variables; low CVH was defined as an LE8 score of 0 to 49, moderate CVH, 50 to 79, and high CVH, 80 to 100.

TD was considered present when the total testosterone level <300ng/dL.

### 3.2 Association of LE8 scores and testosterone deficiency

**Figure 2:**
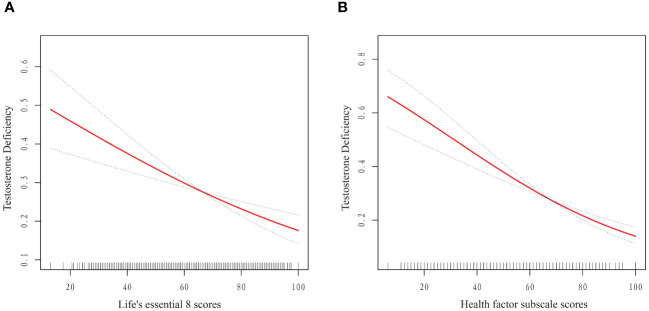
Dose–response relationships between Life’s Essential 8 scores **(A)**, Health Factor scores **(B)** and risk of testosterone deficiency. OR (solid red lines) and 95% CIs (dashed lines) were adjusted for all variables in Model 3 including age, race, marital status, educational level, PIR, BMI, smoking status, alcohol intake, hypertension, diabetes mellitus, CVD, hyperlipidemia, TC, HDL, and UA. (OR, odd ratio; 95%CI, 95% confidence interval; BMI, body mass index; PIR, poverty income ratio; UA, uric acid; TC, total cholesterol; HDL, high-density lipoprotein; CVD, cardiovascular disease.).

As displayed in **Table 2**, weighted logistic regression revealed negative associations between LE8 as well as CVH and TD. The fully adjusted multivariate logistic regression model revealed that the OR for TD was 0.79 (95% CI, 0.71–0.88) for each 10-point increase in LE8 score. And compared with the high CVH group, the ORs of TD were 1.48 (95% CI 1.07–2.05) in the moderate CVH group and 1.90 (95%CI 1.22–2.96) in the low CVH group, respectively. The negative associations showed statistically significant dose-increasing trend (P=0.005). Furthermore, as shown in **Figure 2A**, a linear association was observed between the LE8 score and TD.

**Table 2:** Association of the Life’s Essential 8 scores with testosterone deficiency.

	Non-adjusted model (Model 1)		Minimally adjusted model (Model 2)		Fully adjusted model (Model 3)	
	OR (95%CI)	P	OR (95%CI)	P	OR (95%CI)	P
**LE8 Score** (continuous) Per 10 points increase	0.70(0.66,0.74)	<0.0001	0.68(0.64,0.72)	<0.0001	0.79(0.71,0.88)	<0.001
CVH level						
High CVH (80–100)	Reference		Reference		Reference	
Moderate CVH (50–79)	2.56(1.96,3.35)	<0.0001	2.60(1.97,3.43)	<0.0001	1.48(1.07,2.05)	0.02
Low CVH (0–49	4.80(3.47,6.65)	<0.0001	5.02(3.57,7.05)	<0.0001	1.90(1.22,2.96)	0.01
P for trend	<0.0001		<0.0001		0.005	
**Health behaviors score** (continuous) Per 10 points increase	0.95(0.91,0.99)	0.02	0.93(0.89,0.98)	0.005	0.94(0.88,1.01)	0.08
Classification						
High (80–100)	Reference		Reference		Reference	
Moderate (50–79)	1.38(1.11,1.70)	0.004	1.47(1.18,1.83)	0.001	1.25(1.01,1.54)	0.04
Low (0–49	1.29(1.02,1.62)	0.03	1.41(1.08,1.83)	0.01	1.19(0.86,1.65)	0.28
P for trend	0.011		0.003		0.147	
**Health factors score** (continuous) Per 10 points increase	0.69(0.66,0.72)	<0.0001	0.69(0.66,0.72)	<0.0001	0.74(0.66,0.83)	<0.0001
Classification						
High (80–100)	Reference		Reference		Reference	
Moderate (50–79)	3.32(2.60,4.23)	<0.0001	3.21(2.53,4.08)	<0.0001	1.88(1.36,2.59)	<0.001
Low (0–49	6.79(5.23,8.82)	<0.0001	6.70(5.22,8.61)	<0.0001	2.79(1.79,4.34)	<0.0001
P for trend	<0.0001		<0.0001		<0.0001	

LE8, life’s essential 8; CVH, cardiovascular health; OR, odd ratio; 95%CI, 95% confidence interval; BMI, body mass index; PIR, poverty income ratio; UA, uric acid; TC, total cholesterol; HDL, high-density lipoprotein; CVD, cardiovascular disease.

Model 1 was unadjusted.

Model 2 was adjusted for age, race, marital status, educational level, and PIR.

Model 3 was adjusted for age, race, marital status, educational level, PIR, BMI, smoking status, alcohol intake, hypertension, diabetes mellitus, CVD, hyperlipidemia, TC, HDL, and UA.

### 3.3 Association of health behaviors/health factors with testosterone deficiency

**Table 2** also presented the results of weighted logistic regression of health behavior scores and health factor scores with TD. After multivariable adjustment, no significant associations were found between health behavior score and TD (OR: 0.94, 95% CI 0.88–1.01). Similarly, compared with the high health behavior, the risk of CKD was not significantly different in the moderate and high health behavior groups. On the contrary, the OR for TD was 0.74 (95% CI, 0.66–0.83) for each 10-point increase in health factor score. Additionally, compared with the high health factors group, the ORs of TD were 1.88 (95% CI 1.36–2.59) in the moderate health factors group and 2.79 (95%CI 1.79–4.34) in the low health factors group, respectively (P for trend <0.0001). A linear association was observed between the health factors score and TD (**Figure 2B**). The linear associations between LE8 score and health factor score with TD exhibited similar trends.

### 3.4 Stratified analyses of LE8 with testosterone deficiency

**Figure 3:**
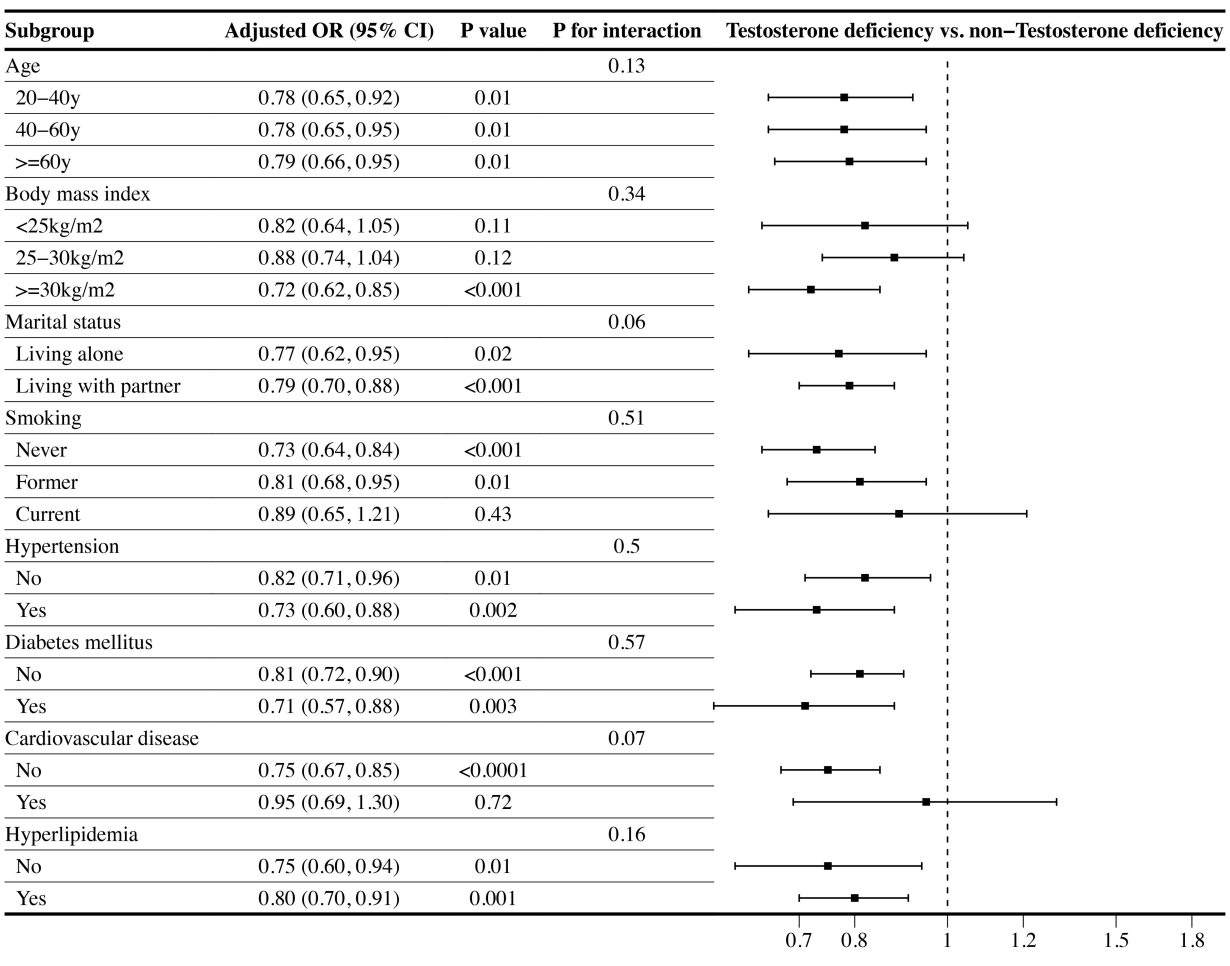
Subgroup analysis of the association of the Life’s Essential 8 scores and the presence of testosterone deficiency (TD). ORs were calculated as per 10 scores increase in LE8 score. Each stratification was adjusted for age, race, marital status, educational level, PIR, BMI, smoking status, alcohol intake, hypertension, diabetes mellitus, CVD, hyperlipidemia, TC, HDL, and UA. (OR, odd ratio; 95%CI, 95% confidence interval; BMI, body mass index; PIR, poverty income ratio; UA, uric acid; TC, total cholesterol; HDL, high-density lipoprotein; CVD, cardiovascular disease.).

Subgroup analyses were performed on the associations between LE8 as well as CVH and TD. As displayed in **Table 3**, the subgroup results stratified by age showed that the negative association between CVH and TD remain stable only in the population over 60 years. Additionally, the low CVH had a higher risk of TD than high CVH in obese subgroup (OR: 2.23, 95% CI 1.09–4.56). Furthermore, the inverse association between CVH and TD remained stable in population with or without diabetes mellitus, and with hyperlipidemia. However, only subgroup population without CVD demonstrated negative association between CVH and TD. No significant interactions were found between CVH and stratified factors in the association with TD (all P for interaction ≥0.05). The **Figure 3** illustrated subgroup analyses of the negative association between LE8 score and TD. LE8 scores were negatively associated with risk of TD in all subgroups except participants with BMI <30 kg/m^2^. Similarly, only participants without CVD displayed negative association between LE8 and TD (OR: 0.75, 95% CI 0.67–0.85). No significant interactions were found between LE8 and stratified factors in the association with TD (all P for interaction ≥0.05).

**Table 3:** Stratified analyses on testosterone deficiency and Life’s Essential 8.

Subgroup analysis	Cardiovascular Health (LE8)					P for trend	P for interaction
	High (80–100)	Moderate (50–79)	P value	Low (0–49)	P value		
**Age**							0.05
20–40y	Reference	1.65(1.04,2.62)	0.03	1.95(0.92,4.11)	0.08	0.03	
40–60y	Reference	1.25(0.77,2.03)	0.35	1.55(0.78,3.10)	0.20	0.21	
≥60y	Reference	1.33(0.75,2.36)	0.31	2.46(1.24,4.89)	0.01	0.01	
**BMI**							0.67
<25kg/m^2^	Reference	1.85(0.94,3.64)	0.07	1.39(0.31,6.17)	0.65	0.08	
25–30kg/m^2^	Reference	1.27(0.84,1.92)	0.24	1.52(0.69,3.31)	0.28	0.19	
≥30kg/m^2^	Reference	1.61(0.79,3.28)	0.18	2.23(1.09,4.56)	0.03	0.02	
**Marital status**							0.08
Living alone	Reference	2.19(0.93,5.19)	0.07	2.24(0.83,6.06)	0.11	0.09	
Living with partner	Reference	1.33(0.98,1.81)	0.06	1.94(1.24,3.02)	0.01	0.01	
**Smoking**							0.68
Never	Reference	1.62(1.06,2.48)	0.03	1.71(0.82,3.57)	0.15	0.03	
Former	Reference	1.21(0.60,2.43)	0.59	1.82(0.89,3.74)	0.10	0.08	
Current	Reference	6.00(0.54,66.20)	0.14	7.45(0.59,93.22)	0.11	0.33	
**Hypertension**							0.52
No	Reference	1.50(1.08,2.10)	0.02	1.54(0.88,2.67)	0.12	0.01	
Yes	Reference	1.57(0.71,3.47)	0.25	2.40(0.94,6.10)	0.07	0.03	
**Diabetes mellitus**							0.15
No	Reference	1.44(1.06,1.97)	0.02	1.99(1.22,3.25)	0.01	0.01	
Yes	Reference	5.04(1.20,21.10)	0.03	6.15(1.43,26.36)	0.02	0.14	
**Cardiovascular disease**							0.10
No	Reference	1.51(1.08,2.11)	0.02	2.23(1.37,3.62)	0.002	0.002	
Yes	Reference	1.40(0.44,4.38)	0.55	1.16(0.30,4.55)	0.82	0.95	
**Hyperlipidemia**							0.23
No	Reference	1.77(0.97,3.24)	0.06	2.26(0.97,5.30)	0.06	0.04	
Yes	Reference	1.31(0.89,1.93)	0.16	1.77(1.04,3.00)	0.04	0.03	

LE8, life’s essential 8; BMI, body mass index; PIR, poverty income ratio; UA, uric acid; TC, total cholesterol; HDL, high-density lipoprotein; CVD, cardiovascular disease; CVH, cardiovascular health.

The subgroup analysis was adjusted for age, race, marital status, educational level, PIR, BMI, smoking status, alcohol intake, hypertension, diabetes mellitus, CVD, hyperlipidemia, TC, HDL, and UA.

## 4 Discussion

To our knowledge, our study is the first to explore the association between LE8 as well as CVH and prevalence of TD, utilizing a nationally representative sample of US male adults from the NHANES database. Our findings indicated a negative and linear association between LE8 score and TD risk. Intriguingly, only health factors subscale displayed a significant negative association with TD risk, when separating the LE8 scores into health behavior and health factor subscale. Subgroup analysis demonstrated that the negative association between CVH and TD remained stable in obese participants (BMI>30 kg/m^2^) or older participants (age>60 years). Furthermore, only participants without CVD demonstrated a consistent negative association between LE8 as well as CVH and risk of TD.

Current researches on the relationship between testosterone levels and CVH predominantly focuses on exploring the impact of testosterone levels on future cardiovascular conditions. An Australia based cohort demonstrated that low testosterone could predict CVD mortality in older men (24). In a cohort in the Central Denmark Region, researchers also revealed strong predictive role of low testosterone for future 5-years risks of cardiovascular outcomes (25). A meta-analysis included 12 community-based studies furtherly confirmed the association between low testosterone level and increased risk of CVD death (26). Collectively, these findings underscore the significance of considering testosterone levels as a potential predictor for adverse cardiovascular events. However, there is significant disagreements in current research results when it comes to supplementing testosterone for patients with TD to reduce the incidence of CVD. Anderson et al. displayed that testosterone replacement to normal level could decrease the major adverse cardiovascular events and death in over 3 years (27). However, a more recent meta-analysis involving 2994 eligible elderly men indicated that testosterone supplementation might increase the risk of CVD, especially in studies without pharmaceutical industry support (28). These discrepancies highlight the complexity of the relationship between testosterone level and CVH. Furthermore, these discrepancies underscore the importance of early identification of TD and prompt intervention, thereby avoiding unnecessary testosterone supplementation.

Consequently, shifting the focus to our pioneering study, we substantiated the inverse relationship between ideal CVH and risk of TD. The CVH was assessed using the LE8 score, a universally recognized and effective indicator associated with CVD (29), non-alcoholic fatty liver disease (30), chronic kidney disease (18), and abdominal aortic calcification (31), among other conditions. Although the underlying mechanisms between LE8 and TD remain unclear, extensive investigations have revealed that development of TD is significantly associated with obesity (32), diabetes (33), hypertension (34), and metabolic syndrome (35), all of which were intrinsic health factors metrics of LE8. Obesity was considered the single most important risk factor for TD. Aromatase, responsible for catalyzing the aromatization of testosterone into estradiol, is predominantly expressed in adipocytes (36). An increase in adipocyte mass enhances aromatase expression. The elevated aromatase, in turn, converts testosterone to estrogen in peripheral adipose tissue, and thus the higher estradiol levels observed in men with obesity (37). Subsequently, the released estrogen exerts negative feedback on GnRH release in the hypothalamus, leading to the inhibition of the HPGA axis (38). Ultimately, this cascade results in a decline in testosterone release within the testes. Another mechanism for obesity-induced TD may be account for leptin, which is a hormone predominantly secreted by white adipose tissue and could regulate the HPGA axis by stimulating GnRH release. However, excessive leptin secretion resulting from obesity can induce leptin resistance in the hypothalamus, consequently suppressing GnRH release and inhibiting testosterone secretion (38). In the context of hypertension, diabetes, and metabolic syndrome, in addition to the potential mechanisms mentioned above, inflammation is a crucial mechanism leading to a decline in testosterone levels. Abnormalities in blood glucose and lipid can promote the production of proinflammatory substances (e.g., IL-6 and TNF-α) from endothelial cells and monocytes (39). These secreted inflammatory factors can, on one hand, directly damage the Leydig cells and even inhibit steroidogenesis by aggravating inflammation and reactive oxygen species production (40). On the other hand, they can also directly induce inflammatory injury in the HPGA (41). Therefore, these findings may mechanistically explain the role of LE8 in the development of TD.

In our results, an interesting finding was that when we divided LE8 scores into health behaviors and health factors subscale, there was no statistically significant association between health behaviors with the risk of TD, which included diet, physical activity, sleep health, and nicotine exposure. One study suggests that low-fat diets may reduce testosterone levels (8), while another study contends that a plant-based diet is unrelated to testosterone levels (42). Previous study displayed that serum testosterone levels in smokers were significantly higher than in nonsmokers (43), an tobacco used could reduce the risk of TD (44). The current conclusions regarding the relationship between sleep duration and TD are inconsistent. Whether a shortened sleep duration leads to a decrease in testosterone levels seems to have some correlation with age and the specific time period of sleep. An animal study suggests that compared to young rats, older rats experience a more pronounced decrease in testosterone levels and slower recovery when subjected to paradoxical sleep deprivation (45). Furthermore, in a subsequent population study where sleep was restricted during the first half of the night and permitted from 04:00–08:00 hours for five nights, no significant change in testosterone was observed (46). These contradictory phenomena may potentially explain the lack of statistical significance between the LE8 health behavior subscale and the risk of TD. However, considering that health behavior factors are relatively easy to modify, further exploration of their relationship with the risk of TD is necessary, which can provide theoretical support for clinicians in advising patients on lifestyle modifications to potentially mitigate the risk of TD.

In the subgroup analysis stratified by CVD, we observed that the negative association between LE8 and TD remained statistically significant only among participants without CVD. Considering the nature of cross-sectional study, this could potentially be explained through reverse causality. For participants with comorbid CVD, there might be a heightened focus on smoking cessation, dietary improvements, increased physical activity, and enhanced sleep (47). Simultaneously, they may also be taking medications to lower blood pressure, glucose, and lipid levels (48). These factors could attenuate the observed relationship between LE8 and TD. Certainly, for a more in-depth exploration of the relationship between the two in individuals with cardiovascular disease, future cohort studies are warranted.

### 4.1 Strengths and limitations

Our study possessed several strengths. Firstly, the study is the first to investigate the associations between LE8 as well as CVH and risk of TD using a large nationally representative sample of US male adults, which ensure higher quality of data and the boarder generalization of findings. Secondly, the dose–response relationships between LE8 and health factor subscale and prevalence of TD were also addressed in our study. Additionally, stratified analyses were performed in different population to evaluate the robustness of the association between LE8 and TD. Therefore, our findings have broader public health implications for the prevention of TD. However, several inherent limitations must be considered seriously when interpreting our findings. First and foremost, the assessments of health behavior metrics relied on self-report questionnaires which are susceptible to recall bias. Secondly, NHANES database did not provide specific symptoms and/or signs associated with TD, leading to the diagnosis of TD solely based on a total testosterone level below 300 ng/dL. Furthermore, the total testosterone level was measured only once for each participant, limiting the ability to diagnose TD based on two separate measurements. So, more clinic-based researches are needed to validate the association between LE8 and functional hypogonadism symptoms. Thirdly, despite controlling for several potential cofounders, the nature of cross-sectional study precludes us from concluding causality and temporality between CVH and TD risk. Finally, only participants aged >20 years were included in our analysis. Overall, while the study provides valuable insights into the association between cardiovascular health and TD in US men, these strengths and limitations should be considered when interpreting the results and designing future research in this area. Moreover, well-designed cohort studies included wider population should be performed in the future.

## 5 Conclusion

In this nationally representative sample of U.S. male adults, LE8 scores and health factor scores were negatively and linearly associated with the lower prevalence of TD. These findings indicated a potential beneficial role of LE8 for promoting testis health. Furthermore, the LE8 may be applied in clinical practice to help male population, identify the risk of TD early and minimize the burden of TD. Further researches are needed in the future to explore the causal relationship and exact mechanism between LE8 and TD.

## Data availability statement

The raw data supporting the conclusions of this article will be made available by the authors, without undue reservation.

## Ethics statement

The studies involving humans were approved by The NCHS Research Ethics Review Committee reviewed and approved the NHANES study protocol (NCHS IRB/ERB Protocol No. #2011-17). All participants signed written informed consent. The studies were conducted in accordance with the local legislation and institutional requirements. The participants provided their written informed consent to participate in this study.

## Author contributions

YM: Conceptualization, Data curation, Formal analysis, Investigation, Methodology, Resources, Supervision, Writing – original draft, Writing – review & editing. NJ: Conceptualization, Formal analysis, Investigation, Methodology, Writing – original draft, Writing – review & editing. BZ: Conceptualization, Formal analysis, Methodology, Software, Supervision, Writing – review & editing. WX: Formal analysis, Funding acquisition, Resources, Supervision, Validation, Visualization, Writing – review & editing. XF: Conceptualization, Data curation, Formal analysis, Funding acquisition, Investigation, Methodology, Project administration, Resources, Software, Supervision, Validation, Visualization, Writing – original draft, Writing – review & editing. RX: Funding acquisition, Investigation, Project administration, Resources, Supervision, Validation, Visualization, Writing – review & editing. DX: Conceptualization, Funding acquisition, Investigation, Methodology, Project administration, Supervision, Validation, Visualization, Writing – review & editing.
