# Supplementary material for: Association between life’s essential 8 and testosterone deficiency in US men: findings from national health and nutrition examination survey (NHANES) 2011–2016
Source: Front Endocrinol (Lausanne). 2024 Jun 24;15:1395576. doi: 10.3389/fendo.2024.1395576 (PMC11228159; doi:10.3389/fendo.2024.1395576)
Supplement: Supplementary file 1 [file Table_1.docx]

**Table S1.** Definition and scoring approach for the American Heart Association’s Life’s Essential 8 score.

| **Domain** | **CVH Metric** | **Measurement** | **Quantification and Scoring of CVH Metric** |
| --- | --- | --- | --- |
| **Health Behaviors** | **Diet** | Healthy Eating Index-2015 diet score percentile | ﻿**Metric:** Quantiles of Healthy Eating Index-2015 |
|  |  |  | ﻿**Scoring (Population):** |
|  |  |  | ﻿**Points Quantile** |
|  |  |  | 100 ﻿≥95th percentile (top/ideal diet) |
|  |  |  | 80 ﻿75th – 94th percentile |
|  |  |  | 50 ﻿50th – 74th percentile |
|  |  |  | 25 ﻿25th – 49th percentile |
|  |  |  | 0 ﻿1st – 24th percentile (bottom/least ideal quartile) |
|  | **Physical activity** | Self-reported minutes of moderate or vigorous physical activity per week | **Metric:** Minutes of moderate (or vigorous) intensity activity per week |
|  |  |  | ﻿**Scoring:** |
|  |  |  | **Points Minutes** |
|  |  |  | 100 ≥150 |
|  |  |  | 90 120-149 |
|  |  |  | 80 90-119 |
|  |  |  | 60 60-89 |
|  |  |  | 40 30-59 |
|  |  |  | 20 1-29 |
|  |  |  | 0 0 |
|  | **Nicotine exposure** | Self-reported use of cigarettes or inhaled nicotine-delivery system | ﻿**Metric:** Combustible tobacco use and/or inhaled NDS use; or secondhand smoke exposure |
|  |  |  | ﻿**Scoring:** |
|  |  |  | **Points** **Status** |
|  |  |  | 100 ﻿Never smoker |
|  |  |  | 75 ﻿Former smoker, quit ≥5 years |
|  |  |  | 50 ﻿Former smoker, quit 1–<5 years |
|  |  |  | 20 ﻿Former smoker, quit <1 year, or currently using inhaled NDS |
|  |  |  | 0 ﻿Current smoker |
|  |  |  | ﻿Subtract 20 points (unless score is 0) for living with active indoor smoker in home |
|  | **Sleep health** | ﻿Self-reported average hours of sleep per night | ﻿**Metric:** Average hours of sleep per night (h) |
|  |  |  | **Scoring:** |
|  |  |  | **Points** **Level** |
|  |  |  | 100 7-<9 |
|  |  |  | 90 9-<10 |
|  |  |  | 70 6-<7 |
|  |  |  | 40 5-<6 or ≥10 |
|  |  |  | 20 4-<5 |
|  |  |  | 0 <4 |
| **Health Factors** | **Body mass index** | Body weight (kg) divided by height squared (m^2^) | ﻿**Metric:** Body mass index (kg/m^2^) |
|  |  |  | **Scoring:** |
|  |  |  | **Points** **Level** |
|  |  |  | 100 <25.0 |
|  |  |  | 70 25.0-29.9 |
|  |  |  | 30 30.0-34.9 |
|  |  |  | 15 35-39.9 |
|  |  |  | 0 ≥40.0 |
|  | **Blood lipids** | Plasma total and HDL-cholesterol with calculation of non-HDL-cholesterol | ﻿**Metric:** Non-HDL-cholesterol (mg/dL) |
|  |  |  | **Scoring:** |
|  |  |  | **Points** **Level** |
|  |  |  | 100 <130 |
|  |  |  | 60 130-159 |
|  |  |  | 40 160-189 |
|  |  |  | 20 190-219 |
|  |  |  | 0 >220 |
|  |  |  | ﻿If drug-treated level, subtract 20 points |
|  | **Blood glucose** | Fasting blood glucose or casual hemoglobin A1c | ﻿**Metric:** Fasting blood glucose (mg/dL) or Hemoglobin A1c (%) |
|  |  |  | **Scoring:** |
|  |  |  | **Points** **Level** |
|  |  |  | 100 ﻿No history of diabetes and FBG <100 (or HbA1c < 5.7) |
|  |  |  | 60 ﻿No diabetes and FBG 100 – 125 (or HbA1c 5.7 – 6.4) (Prediabetes) |
|  |  |  | 40 ﻿Diabetes with HbA1c <7.0 |
|  |  |  | 30 ﻿Diabetes with HbA1c 7.0 – 7.9 |
|  |  |  | 20 ﻿Diabetes with HbA1c 8.0 – 8.9 |
|  |  |  | 10 ﻿Diabetes with HbA1c 9.0 – 9.9 |
|  |  |  | 0 ﻿Diabetes with HbA1c ≥10.0 |
|  | **Blood pressure** | Appropriately measured systolic and diastolic blood pressure | ﻿**Metric:** Systolic and diastolic blood pressure (mm Hg) |
|  |  |  | **Scoring:** |
|  |  |  | **Points** **Level** |
|  |  |  | 100 ﻿<120/<80 (Optimal) |
|  |  |  | 75 ﻿120 – 129/<80 (Elevated) |
|  |  |  | 50 ﻿130 – 139 or 80 – 89 (Stage I HTN) |
|  |  |  | 25 ﻿140 – 159 or 90 – 99 |
|  |  |  | 0 ﻿≥160 or ≥100 |
|  |  |  | ﻿Subtract 20 points if treated level |

**Abbreviation**

CVH, cardiovascular health; NDS, nicotine-delivery systems; HDL, high-density lipoprotein; FBG, fasting blood glucose; HbA1c, glycated hemoglobin; HTN, hypertension.

**Reference**

1. Lloyd-Jones DM, Allen NB, Anderson CAM, et al. Life's Essential 8: Updating and Enhancing the American Heart Association's Construct of Cardiovascular Health: A Presidential Advisory From the American Heart Association. Circulation. Aug 2 2022;146(5): e18-e43.

2. Lloyd-Jones DM, Ning H, Labarthe D, et al. Status of Cardiovascular Health in US Adults and Children Using the American Heart Association's New "Life's Essential 8" Metrics: Prevalence Estimates From the National Health and Nutrition Examination Survey (NHANES), 2013 Through 2018. Circulation. Sep 13 2022;146(11):822-835
